# Inhibition of ALK1 signaling with dalantercept combined with VEGFR TKI leads to tumor stasis in renal cell carcinoma

**DOI:** 10.18632/oncotarget.9621

**Published:** 2016-05-26

**Authors:** Xiaoen Wang, Nicolas Solban, Prateek Khanna, Marcella Callea, Jiaxi Song, David C. Alsop, R. Scott Pearsall, Michael B. Atkins, James W. Mier, Sabina Signoretti, Marat Alimzhanov, Ravi Kumar, Manoj K. Bhasin, Rupal S. Bhatt

**Affiliations:** ^1^ Division of Hematology-Oncology and Cancer Biology, Beth Israel Deaconess Medical Center, Harvard Medical School, Boston, MA, USA; ^2^ Department of Radiology, Beth Israel Deaconess Medical Center, Harvard Medical School, Boston, MA, USA; ^3^ Acceleron Pharma, Inc., Cambridge, MA, USA; ^4^ Department of Pathology, Brigham and Women's Hospital, Boston, MA, USA; ^5^ Departments of Oncology and Medicine, Georgetown Lombardi Comprehensive Cancer Center, Washington, DC, USA; ^6^ Division of Interdisciplinary Medicine & Biotechnology, and Genomics, Proteomics, Bioinformatics and Systems Biology Center, Department of Medicine, Beth Israel Deaconess Medical Center, Boston, MA, USA

**Keywords:** renal cell carcinoma, anti-angiogenic therapy, ALK-1, VEGF, dalantercept

## Abstract

Treatment of metastatic renal cell carcinoma (mRCC) with agents that block signaling through vascular endothelial growth factor receptor 2 (VEGFR2) induces disease regression or stabilization in some patients; however, these responses tend to be short-lived. Therefore, development of combination therapies that can extend the efficacy of VEGFR antagonists in mRCC remains a priority.

We studied murine xenograft models of RCC that become refractory to treatment with the VEGFR tyrosine kinase inhibitor (TKI) sunitinib. Dalantercept is a novel antagonist of Activin receptor-like kinase 1 (ALK1)/Bone morphogenetic protein (BMP) 9 signaling. Dalantercept inhibited growth in the murine A498 xenograft model which correlated with hyperdilation of the tumor vasculature and an increase in tumor hypoxia. When combined with sunitinib, dalantercept induced tumor necrosis and prevented tumor regrowth and revascularization typically seen with sunitinib monotherapy in two RCC models. Combination therapy led to significant downregulation of angiogenic genes as well as downregulation of endothelial specific gene expression particularly of the Notch signaling pathway.

We demonstrate that simultaneous targeting of molecules that control distinct phases of angiogenesis, such as ALK1 and VEGFR, is a valid strategy for treatment of mRCC. At the molecular level, combination therapy leads to downregulation of Notch signaling.

## INTRODUCTION

An increased understanding of the molecular biology of RCC has led to the identification of angiogenesis as a key contributor to its pathogenesis. Alterations in the Von Hippel Lindau (VHL) gene, hypoxia-inducible factor 1- alpha (HIF1-α) accumulation and vascular endothelial growth factor (VEGF) overexpression have been documented in the majority of clear cell RCC. Multiple antagonists of the VEGF signaling pathway such as the multikinase inhibitors sunitinib, pazopanib, axitinib and sorafenib and the monoclonal antibody to VEGFA, bevacizumab, are approved for treatment of patients with advanced RCC [1–4]. While each of these anti-VEGF strategies has shown clinical benefit in the majority of patients, many patients are either resistant to such therapy or more commonly acquire resistance to these drugs within a year of treatment initiation. Therefore, targeting additional angiogenic signaling pathways may represent a promising therapeutic approach for patients with advanced RCC.

We have previously shown in a mouse model of RCC that resistance to VEGFR inhibitors is accompanied by reestablishment of tumor vasculature [5]. Tumors treated with sunitinib showed a brief period of tumor stabilization and decreased blood flow as measured by arterial spin labeled magnetic resonance imaging (ASL MRI) [5]. Subsequently, despite continued sunitinib administration, tumors showed a resumption of blood flow (albeit to a lesser extent than vehicle treated tumors) and tumor growth. We have shown by IHC and ASL MRI, that the development of sunitinib-resistant vessels is inhibited by supplemental activation of endogenous angiostatic pathways such as CXCL9 [5]. Based on these findings, we hypothesize that optimal antiangiogenic regimens will include combinations of agents targeting different steps in tumor angiogenesis.

Given the complexity of the neoangiogenic process, it is likely that simultaneous inhibition of multiple regulatory pathways including VEGF will increase the magnitude and duration of antitumor effects in RCC. Members of the TGF-beta superfamily have been implicated as important regulators of angiogenesis during embryonic development [6–8]. ALK1 and endoglin (ENG) are receptors in the TGF-beta superfamily of signaling molecules known to be expressed on actively forming arterial endothelial cells [9,10]. The superfamily ligands bone morphogenetic protein (BMP) 9 and 10 bind with high affinity to a heteromeric complex consisting of ALK1, a cognate superfamily type II receptor, and ENG, an accessory receptor, to induce specific serine/threonine kinase activity and phosphorylation of regulatory proteins (SMAD 1/5/8) which translocate to the nucleus and activate transcription of target genes [6,9,11–13].

Evidence for ALK1 participation in vascular formation came from the discovery that mutations in the human ACVRL1 gene (encodes ALK1 protein) are the cause of hereditary hemorrhagic telangiectasia (HHT) type 2 [14]. Mutations in the human endoglin (ENG, CD105) gene were found to be responsible for HHT type 1, thus indicating the importance of this signaling pathway in vascular formation. [15]. Patients with HHT exhibit abnormal vessel development, including arteriovenous malformations (AVMs) in which intervening capillary beds are absent. Genetic knockout of either the ACVRL1 or ENG genes in mice leads to embryonic lethality due to defective angiogenesis. Heterozygous mice carrying a single allele of ACVR1 or ENG develop HHT-like symptoms including the development of telangiectasias [16,17]. Whereas VEGF is known to play a key role in initiation of angiogenesis, ALK1 pathway has been shown to regulate the maturation phase of angiogenesis [18].

Dalantercept is a fully-human, recombinant fusion protein produced by linking the extracellular domain of the human ALK1 receptor to the Fc portion of human immunoglobulin G1 (ALK1-Fc) [12]. Dalantercept functions as a selective trap for BMP9/BMP10, and we and others have shown that blocking ALK1 signaling with ALK1-Fc results in defective development of vascular and lymphatic networks *in vivo* [6,7,12]. Treatment with ALK1-Fc suppressed tumor progression and decreased tumor vasculature in a RIP1-Tag2 transgenic model of pancreatic islet cell cancer [19]. Interestingly, similar to ALK1-Fc protein, soluble endoglin-Fc was found to bind selectively to BMP9/BMP10 and to effectively inhibit both angiogenesis and tumor xenograft growth *in vivo* [11].

In the present study we show that combined inhibition of ALK1 and VEGFR pathways has profound effects on tumor angiogenesis. The mechanism of action of the combination treatment is likely in part due to dysregulation of interconnected VEGF/VEGFR, BMP/ALK1 and Dll4/Notch signaling pathways, which interferes with the development of acquired resistance to VEGFR TKI. Thus, combined antagonism of the ALK1 and VEGFR pathways is a promising novel therapeutic option for patients with advanced RCC.

## RESULTS

### Treatment with dalantercept alters tumor vascular network, increases tumor hypoxia and delays tumor growth

**Figure 1:**
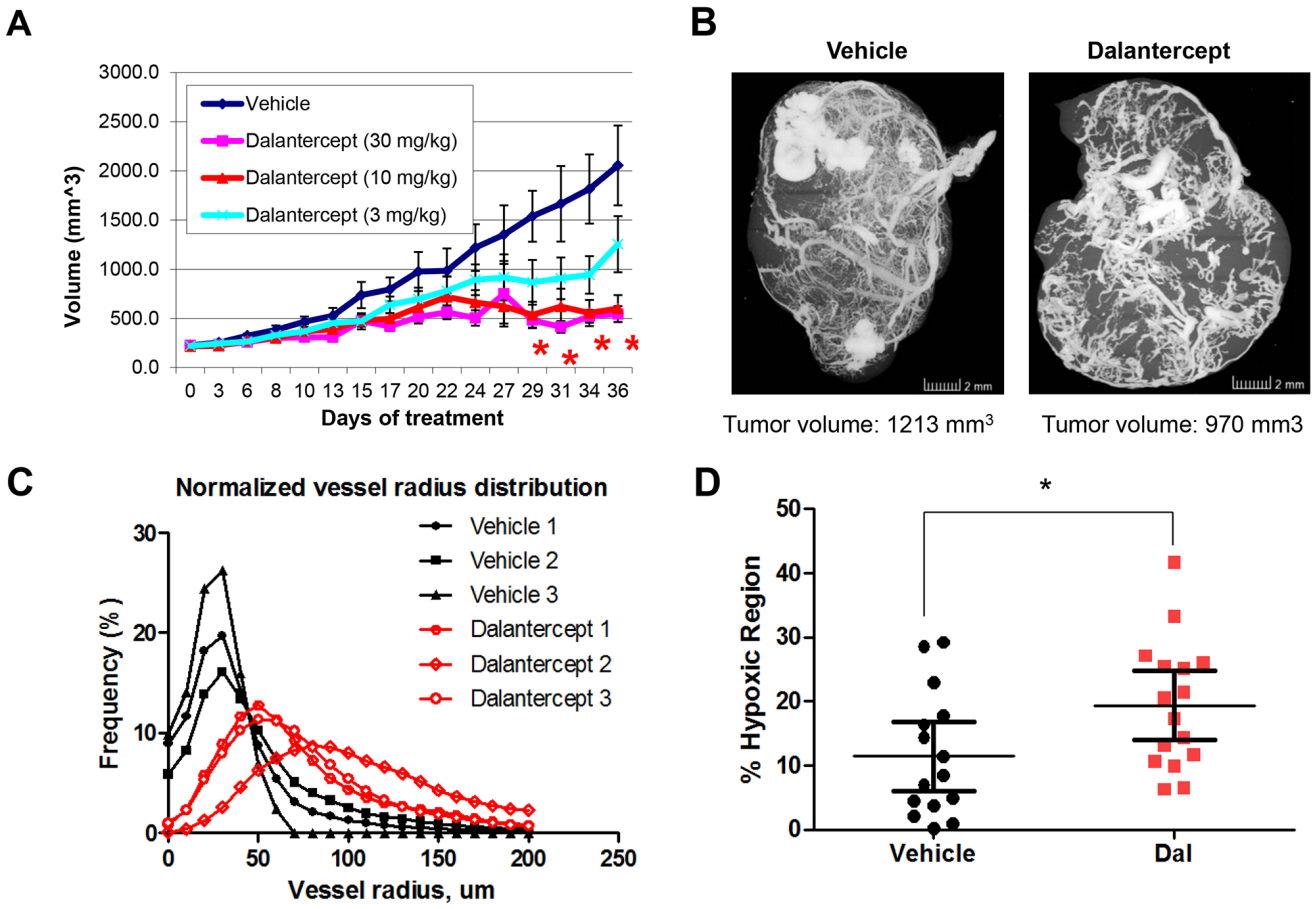
Dalantercept slows RCC tumor growth and affects tumor vasculature *in vivo* **A.** Growth curves depicting tumor volume of A498 derived RCC xenografts treated with vehicle, dalantercept (3mg/kg), dalantercept (10mg/kg) and dalantercept (30mg/kg). **B.** Representative images of tumor vascular networks after treatment with either vehicle or dalantercept (10 mg/kg, i.p., tiw) for 3 weeks. Perfusion with Microfil, acquisition and analysis of uCT images is described in Materials and methods. **C.** Normalized frequency distribution of blood vessel radius calculated from the 3D vascular network models (data from individual tumors, n=3 per group). Blood vessels were binned into three groups according to the vessel radius (<50um, 50-100 um and >100 um) and the frequency of vessels in each group was calculated, P<0.05. **D.** Quantification of hypoxic areas in tumor tissue using hypoxic probe EF5 and immunohistochemistry shows more tumor hypoxia in dalantercept treated tumors as compared to the vehicle treated tumors (*P<0.033).

Treatment with dalantercept delayed growth of A498 human RCC xenograft tumors in a dose-dependent manner with both 10 mg/kg and 30 mg/kg doses showing statistically significant effects on the tumor growth while 3mg/kg showed only a modest effect (Figure 1A). Based on these data, the 10 mg/kg dose of dalantercept was chosen for combination studies with the VEGFR TKI sunitinib (Figure 1A).

To view the *in vivo* treatment-induced changes in the tumor vascular network, we perfused dalantercept-treated and control mice with the Microfil imaging reagent. Three-dimensional reconstruction of the tumor vascular network revealed profound aberrations in the network organization in dalantercept-treated tumors (Figure 1B). Large, dilated blood vessels were prominent in the dalantercept-treated tumors while the typical tree-like branching pattern was missing. Average vessel radius increased from 30 μm in the control tumors to ~60 μm in dalantercept treated tumors, which correlated with an overall shift in the distribution of vessel size toward larger vessels (Figure 1B). The frequency of Microfil-perfused small blood vessels (<50 um radius) was dramatically reduced in dalantercept treated tumors (22% vs 74% in control group), whereas the frequency of large vessels (>50 um or >100 um radius) was correspondingly increased (Figure 1B, 1C).

This phenomenon resembles vascular remodeling and vessel dilation occurring upon formation of arteriovenous malformations (AVMs) in ALK1-deficient blood vessels in a mouse model of HHT [20]. Development of such AVMs in HHT leads to abnormal high-velocity, turbulent arterial blood flow and an elevation of oxygen saturation levels in the venous vessels. Thus we reasoned that it was likely that AVM formation was also taking place in A498 tumors treated with dalantercept. Tumor vascular networks compromised by the AVMs would be less efficient in the delivery of oxygen and nutrients to tumor cells. To test this hypothesis we quantified hypoxic areas in the tumor tissues using the hypoxia probe, EF5 [21]. In line with this hypothesis, immunohistochemical analysis of EF5-positive areas in A498 tumors treated with either vehicle or dalantercept for 2 weeks revealed more extensive tumor hypoxia in dalantercept treated tumors (P<0.033) (Figure 1D).

### Dalantercept combined with sunitinib shows durable tumor stasis by preventing resumption of tumor blood flow in human RCC xenograft models

**Figure 2:**
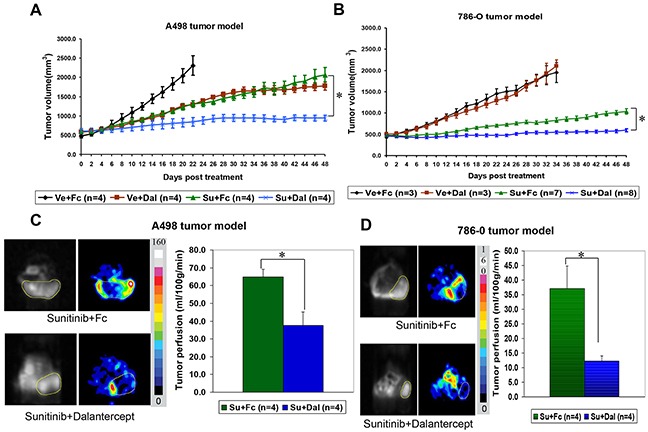
Combination of dalantercept with sunitinib leads to durable tumor growth inhibition in RCC xenograft models A498 tumor-bearing mice **A.** or 786-0 tumor-bearing mice **B.** were randomized into four groups and treated with vehicle and 10 mg/kg control Fc protein (black line); vehicle and 10 mg/kg dalantercept (brown line); 40 mg/kg sunitinib and 10 mg/kg control Fc (green line); or combination of 40 mg/kg sunitinib and 10 mg/kg dalantercept (blue line). Plotted are average tumor volumes +/− SE. Tumor blood flow was measured by ASL MRI in A498 xenograft bearing animals on the day of sacrifice (roughly day 50 of drug treatment). Representative images of A498 **C.** or 786-O **D.** tumors treated with either sunitinib + control Fc or sunitinib + dalantercept are shown. Calculated tumor blood flow was color coded according to the scale from 0 to 160 ml/100g/min. Day 50 tumor blood flow averages (+/−SE) from animals treated with sunitinib + control Fc protein versus combination of sunitinib + dalantercept, *P<0.05.

Next we wanted to explore if combination treatment of dalantercept and a VEGFR antagonist, TKI sunitinib, could provide any additional benefit over sunitinib therapy alone. Treatment with either sunitinib (Su) or dalantercept (Dal) alone slowed A498 tumor growth (Figure 2A), (comparison of tumor volumes on day 22, vehicle 2310.3 ± 251.9 mm^3^ vs Su 1308.3 ± 88.1 mm^3^; P=0.013; and vehicle vs Dal 1290.1 ± 16.7mm^3^; P=0.009). Combination of the two agents led to profound tumor growth inhibition for up to 7 weeks with continuous dosing (Figure 2A), (Su + Dal 944.4 ± 75.4mm^3^ vs Su 2068.8 ± 184.4mm^3^; P=0.003). This combination regimen was also tested in the 786-O RCC xenograft model. While dalantercept monotherapy was not able to inhibit tumor growth in the 786-O model, the combination of dalantercept and sunitinib led to greater suppression of tumor growth as compared with sunitinib monotherapy alone and resulted in durable tumor stasis (Figure 2B), (tumor volume, ~day 34 Su + Dal 548.7 ± 35.5 mm^3^ vs Su 831.0 ± 53.7mm^3^; P=0.0007; and ~Day 48 Su + Dal 596.9 ± 40.5 mm^3^ vs Su 1039.1 ± 63.0 mm^3^; P=0.0001).

Previously, in both A498 and 786-O models, treatment with sunitinib led to a significant reduction in tumor blood flow as early as 3 days after treatment initiation, followed by partial restoration of tumor blood flow after several weeks of chronic dosing [5]. We performed serial ASL MRI imaging to monitor changes in tumor blood flow in relation to the drug treatment in both tumor models. As expected, we observed resumption of tumor blood flow in the A498 model after longterm treatment with sunitinib (Supplementary Figure S1A). In contrast, combination treatment with dalantercept plus sunitinib in A498 tumor-bearing mice showed reduced blood flow compared with sunitinib alone evident on day 50 after treatment initiation (Figure 2C), (Su 64.9 ± 4.2ml/100g/min vs Su + Dal 37.6 ± 7.5ml/100g/min; P=0.02). A similar effect of the combination treatment on tumor blood flow was observed in the 786-O xenograft model (Figure 2D). On day 48, residual tumor blood flow was significantly reduced in the combined-treatment cohort (12.3 ± 1.7ml/100g/min) compared to the sunitinib-only group (37.1 ± 15.6ml/100g/min; P=0.02). Consistent with the Microfil results and the known role of ALK1 on the vasculature, dalantercept monotherapy led to an increase in tumor blood flow (Supplementary Figure S1B).

**Figure 3:**
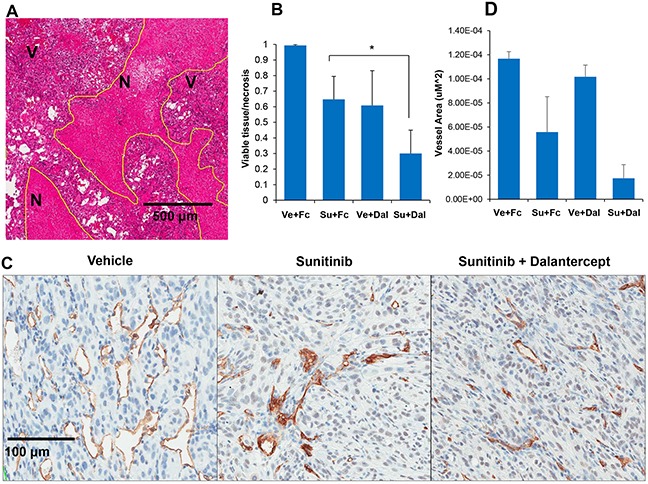
Combination of sunitinib and dalantercept induces tumor necrosis and loss of tumor vasculature **A.** A498 tumors treated with vehicle, Vehicle + dalantercept, sunitinib + control Fc or sunitinib + dalantercept, were stained with hematoxylin and eosin and areas of necrosis and viable tumor were outlined (shown at 5x magnification). **B.** Quantitation of viable tumor/necrosis was performed. (Vehicle vs Su P=0.058; Vehicle vs Dal P=0.04; Vehicle vs Su + Dal P=0.0007; Su vs Su + Dal P=0.038; Dal vs Su + Dal P=0.09. **C.** Immunohistochemistry of CD34 expression in tumors treated with vehicle, sunitinib, and sunitinib plus dalantercept combination. **D.** Quantification of area enclosed by CD 34 positive stain representative of blood vessel area (Vehicle vs Su P=0.005; Vehicle vs Su + Dal P=0.0001; Su vs Dal P=0.03; Su vs Su + Dal P=0.05; Dal vs Su + Dal P=0.0005)

To further assess the effect of dalantercept and sunitinib on tumors, we compared tumor necrosis and microvessel density (MVD) in the treatment arms in the A498 tumor model. At the time of the ASL MRI (day 48-50), viable tumor and necrosis were quantified. The combination of sunitinib and dalantercept yielded a higher ratio of necrosis/viable tumor than either treatment alone (Figure 3A, 3B), (Su + Dal vs Vehicle: P=0.0005; Su + Dal vs Dal: P=0.07; Su + Dal vs Su: P=0.01; Su vs Vehicle: P=0.01; Dal vs Vehicle: P=0.04).

We also performed IHC to assess for the effects of combination treatment on tumor vasculature (Figure 3C). Tumors treated with sunitinib showed a decrease in MVD. Combination therapy decreased MVD to a greater extent than individual therapies (Figure 3C, 3D). However, the comparison of sunitinib vs combination did not reach statistical significance (P=0.05) likely due to the fact that the amount of viable tumor remaining for MVD analysis was extremely small in the combination treatment group and sunitinib treated tumors had low MVD.

### Combination of dalantercept and sunitinib suppresses growth of A498 tumors that progressed on sunitinib monotherapy

**Figure 4:**
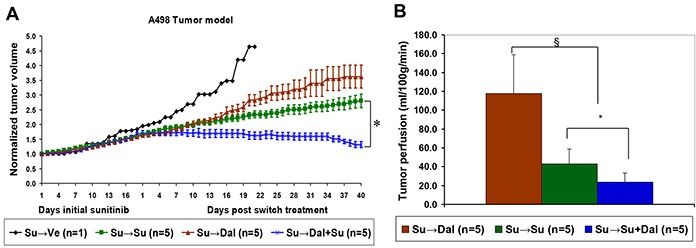
Combination of dalantercept with sunitinib slows tumor growth and further reduces tumor blood flow in sunitinib resistant tumors **A.** A498 tumor-bearing mice were treated with sunitinib (40 mg/kg) until the tumors showed a 2 mm increase in long axis. After a 2 day drug holiday, animals were given vehicle (black line), sunitinib at (40 mg/kg, green line), dalantercept (10 mg/kg, brown line) or the combination of both drugs (blue line). Average tumor volume normalized to the initial average tumor volume for each group is shown. **B.** tumor perfusion was evaluated by ASL MRI on day 40 after the treatment change and average tumor perfusion is plotted in the graph. Su switched to the combination of Su + Dal vs Su continuous; * P=0.047. Su switched to Dal vs Su continuous; § P=0.006.

We then explored the efficacy of combination therapy in the context of sunitinib-refractory RCC. Animals with established A498 tumors were treated with sunitinib until tumor diameter increased by 2 mm (the smallest measurable size increase). At that point, treatment was stopped for 2 days and mice were randomized into three groups: one group continued to receive sunitinib, the second group was switched to dalantercept treatment and the third group received both sunitinib and dalantercept. The combination treatment showed a significant delay of tumor growth compared to either dalantercept alone or continued sunitinib alone (Figure 4A), (normalized tumor volumes on day 40 post treatment switch, Su switched to Dal: 3.6 ± 0.4 vs Su switched to Su + Dal: 1.3 ± 0.1; P=0.008; Su continued: 2.8 ± 0.2 vs Su switched to Su + Dal: 1.3 ± 0.1; P=0.007). This delay in tumor growth was associated with a further decrease in tumor blood flow after combination treatment vs continued sunitinib treatment (Su switched to Su + Dal: 23.7 ± 9.8ml/100g/min vs Su continued: 43.3 ± 15.8ml/100g/min; P=0.046). The switch to dalantercept from sunitinib treatment led to increased tumor blood flow compared to continuous sunitinib treatment (Su switched to Dal: 117.9 ± 41.4ml/100g/min vs Su continued: 43.3 ± 15.8ml/100g/min; P=0.006), as evaluated by ASL MRI on day 40 post switch (Figure 4B).

### Unsupervised analysis of RNA sequencing data shows differential gene expression after ALK1 and VEGFR inhibition

To understand the mechanism underlying combination therapy, RNA sequencing of treated tumors was performed. Mice harboring A498 tumors were treated with vehicle, sunitinib, dalantercept or combination (sunitinib + dalantercept) and tumors were harvested for RNA sequencing at 30 days or when tumors reached 20mm in long axis. High quality RNA sequencing data was aligned against mouse and human genomes to understand the effects of various treatments on host and tumor transcriptomes.

The sequencing reads achieved on average ~79.9% and ~15.2% alignments against tumor and host genomes, respectively. The low alignment against the mouse genome was expected as sequencing was performed on human xenograft tumors and majority of the specimen consisted of human tumor cells with a smaller amount of mouse stromal tissue. The alignments against the mouse genome could represent this host stromal tissue or merely cross-reactivity between mouse and human genomes. For secondary validation of the gene expression changes, we performed qRT-PCR on multiple host significantly differentially expressed genes (Supplementary Figure S2). A significant correlation was observed between RNA-Seq and qRT-PCR based gene expression.

The unsupervised analysis using PCA demonstrated a good separation between untreated and treated samples along primary component (PC) 1. PCA showed that samples treated with combination (sunitinib + dalantercept) and monotherapy (sunitinib or dalantercept) formed separate groups indicating differential transcriptome changes (Supplementary Figure S3).

**Figure 5:**
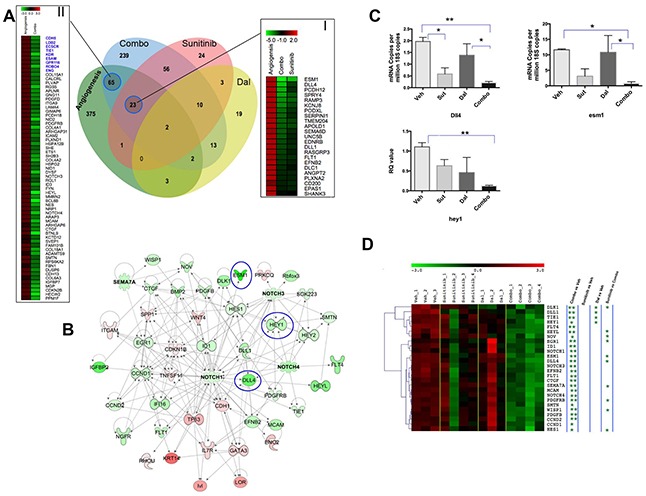
Comparative analysis of tumor angiogenesis signature and RCC xenograft host transcriptome alterations due to sunitinib, dalantercept and combination therapies **A.** Comparative analysis of tumor angiogenesis upregulated genes (Green Circle) with genes that are significantly downregulated with sunitinib (Red Circle), dalantercept (Yellow Circle) and combination therapy (Blue Circle). Combination therapy significantly downregulated ~20% of the upregulated genes (i.e. 92 genes) from the primary tumor angiogenesis signature. Panel I depicts fold change based heatmap of 23 angiogenesis related genes that are downregulated both due to sunitinib and combination therapy. Panel II shows the heatmap of 65 angiogenesis related genes that are only significantly downregulated by combination therapy but not by monotherapy. The panel consists of 9 out of top 20 angiogenesis related genes (shown in Blue) as identified by Masiero et al [22]. **B.** Key regulatory molecules associated with combination treatment. Heatmap depicts key regulatory genes that are specifically inhibited or activated by combinationtreatment. Magnitude of activation and inhibition is calculated on the basis of Z-score and shown using pseudocolor scale (−3 to 3) with red color denoting activated and green color denoting inhibited key regulators. An interactive network of combo effected key regulator and target genes are also shown. The intensity of node color indicates the degree of up-regulation (red) and down-regulation (green) by combination treatment compared to untreated samples. **C.** Validation of Notch signaling target genes altered by combination treatment. RT-PCR was performed on vehicle, sunitinib, dalantercept and combination treated tumors in triplicate with at least two tumors per arm (n=2-3 tumors per arm). Graph shows mean ± SEM of relative mRNA levels after normalization to 18S copies. *, P< 0.05, **, P<0.01. **D.** Combination therapy significantly (P<0.01) downregulated expression of more than 2 dozen endothelial specific genes. The analysis was performed after normalizing the transcript expression to the expression of VWF, an endothelial specific gene. Shown is the heatmap of endothelial genes significantly downregulated by combination therapy. The columns represent samples and the rows represent genes. Gene expression is shown with a pseudocolor scale (−3 to 3) with red color denoting increase and green color denoting decrease in gene expression. The significance of downregulation of endothelial genes in different comparisons is shown by asterisk (** P<0.001, * P<0.05).

We then focused on the “host” genes, which were downregulated by either dalantercept, sunitinib or the combination and compared those to a previously reported angiogenic/Endothelial Cell meta-signature developed using several human cancers including RCC [22]. As shown in Figure 5A, of the 471 upregulated genes from the angiogenesis meta-signature, 92 (~20%) genes were significantly downregulated (Absolute Fold Change ≥2 and FDR P value <0.05) in the combination treatment group. Of the 92 genes, 23 genes were commonly downregulated by both sunitinib monotherapy as well as combination therapy (Figure 5A, Panel I), and 65 genes were significantly affected by the combination treatment only but not by monotherapy (Figure 5A, Panel II). Nine out of the top 20 genes in the angiogenesis meta-signature were significantly downregulated by the combination treatment but not by sunitinib or dalantercept monotherapy, (Figure 5A, Panel II, blue highlighted) suggesting that combination of sunitinib and dalantercept has a more profound effect on tumor angiogenesis than either agent alone.

### Combination therapy leads to downregulation of the Notch pathway

To identify the key pathways and regulators affected by the combination treatment we performed a systems biology oriented analysis of the gene expression changes. Interestingly, Notch signaling emerged as one of the top pathways downregulated specifically in the combination treatment group. The Notch/DLL4 pathway limits vascular sprouting during angiogenesis leading to maturation of the vascular bed. The analysis identified Notch 1 as a central node of the interactive map with multiple members of the Notch family (i.e. Notch1, Notch 3, Notch 4) significantly downregulated by combination treatment (Figure 5B). Expression of Notch pathway signal transducing molecules, such as Hey1/2 and HES1 was also affected. The Notch ligand DLL4 and the endothelial tip cell marker ESM1 were affected both by sunitinib monotherapy as well as combination treatment but more profoundly by the latter. Using qRT-PCR, we further validated significant downregulation of some of these key genes (ESM1, DLL4, HEY1) after combination therapy (Figure 5C).

Reduced expression of genes involved in tumor angiogenesis may reflect general loss of endothelial cells due to the drug treatment. To take this into account, we normalized the expression of angiogenic genes from our murine gene expression dataset to the expression of an endothelial cell specific gene, Von Willebrand factor (VWF). Normalized expression of multiple members of the Notch signaling pathway was still significantly downregulated in the combination group in contrast to sunitinib or dalantercept monotherapy groups (Figure 5D).

### BMP9 is expressed in human RCC

**Figure 6:**
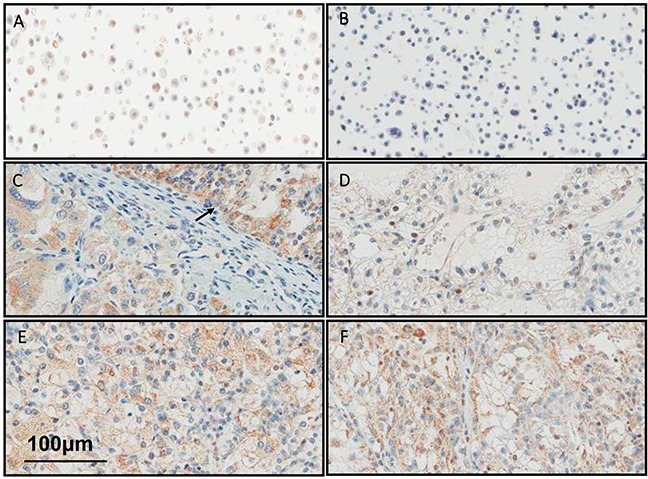
BMP9 is expressed in human RCC Representative images of FFPE samples immunostained with anti-BMP9 antibody are shown. **A.** Positive and **B.** Negative CHO cell line controls were used for antibody optimization. **C.** Positive primary clear cell RCC. Note the presence of positive tumor cells both in Fuhrman nuclear grade 2 area (arrow) and in Fuhrman nuclear grade 2 area, **D.** Negative primary ccRCC, **E.** Positive primary ccRCC and **F.** Corresponding positive lung metastasis.

Previously, ALK1 expression was detected by IHC in the vasculature of most human tumors, including clear cell RCC [23]. Here we assessed BMP9 expression in cases of primary clear cell RCC to assess expression of the ALK1 ligand in human tumor cells (Figure 6). Positive cytoplasmic BMP9 expression in tumor cells was observed in 10 out of 12 (83%) primary clear cell RCC nephrectomy specimens. In one case, the corresponding lung metastasis was also available and BMP9 was observed to be expressed in both the primary tumor and in the metastasis.

## DISCUSSION

Angiogenesis involves a balance of multiple pathways exerting effects on distinct coordinated steps. The ALK1 signaling pathway plays a major role in regulation of normal vasculogenesis. ALK1 and its co-receptor ENG are co-expressed on the surface of endothelial cells during active angiogenesis, and a mutation in either one results in hereditary hemorrhagic telangiectasia (HHT), an autosomal dominant vascular dysplasia syndrome [24,25]. BMP9 and BMP10 are circulating ligands which induce activation of the receptor complex and are thought to play a key role in the maturational phase of the vascular and lymphatic network during embryonic development [6,7,18,26]. We have previously reported that both ALK1-Fc and ENG-Fc fusion proteins selectively bind BMP9 and BMP10 and display single agent anti-angiogenic and anti-tumor effects in mouse models [11,12,19].

Here we used xenograft models of RCC to show that treatment with dalantercept led to profound changes in the tumor vascular network typified by significant reduction in frequency of small blood vessels and an increase in large, dilated vessels (Figure 1B, 1C). The observed changes in the tumor vascular network are consistent with the known phenotype of HHT vessels and with previous results in a mouse model of conditional ALK1 inactivation, in which abnormal arterio-venous junctions appear, leading to shunting of local blood circulation and hyperdilation of veins [17]. However, the blood flow of small vessels and effective delivery of oxygen to tumor cells was significantly decreased under such conditions as revealed by increase in hypoxic areas in the tumors from dalantercept-treated animals (Figure 1D).

This is the first study to demonstrate that combination of the VEGFR inhibitor sunitinib with dalantercept leads to prolonged tumor stasis in human RCC xenograft models. Moreover, the combination of dalantercept and sunitinib was also effective in inhibiting growth of A498 tumors that became refractory to sunitinib monotherapy. The tumor growth inhibition effect of the combination treatment correlated with further decrease in tumor vasculature, tumor perfusion and an increase in tumor necrosis at the end of treatment, compared to sunitinib monotherapy. These data have led to clinical testing of the combination of a VEGFR TKI (axitinib) and dalantercept.

Our analysis of the murine transcriptome of treated A498 xenografts revealed a significant overlap between genes downregulated by either sunitinib monotherapy or the combination treatment and the previously published angiogenic meta-signature (Figure 5A) [22]. Among the most significantly affected genes, we found several members of the Notch family as well Notch signal transducing molecules (Figure 5B). By normalizing gene expression to the endothelial specific gene, VWF, we showed that reduced expression of multiple members of Notch pathway was not simply due to loss of endothelial cells in the treated tumors. It appears that one of the potential mechanisms by which combined VEGFR and ALK1 inhibition may delay angiogenic escape in RCC is through downregulation of the Notch signaling pathway (Figure 5D). ALK1 and Notch pathways have recently been shown to work synergistically in stalk endothelial cells to repress tip cell formation and sprouting [27]. Moreover, DLL4 blockade has been shown to improve the anti-tumor effects of VEGF inhibition in xenograft models [28]. We hypothesize that hypersprouting and the disorganized, immature vascular network induced by ALK1/Notch inhibition is primed for optimal destruction in response to VEGF pathway inhibition.

Due to technical reasons, we were unable to detect BMP9 expression in murine tumors, however we did detect expression in human RCC specimens. It is intriguing that our analysis of a small number of tumor samples from primary RCC showed the majority (10 out 12) of the tumors expressing BMP9 at moderate to high levels. It remains to be determined if tumor BMP9 expression correlates with responses to dalantercept in the clinic.

Several ALK1 pathway antagonists are under current investigation in clinical trials. Dalantercept and antibodies directed against ALK1 (Pfizer, PF-03446962) and ENG (Tracon, TRC105) have all shown antitumor activity in the setting of VEGF pathway inhibition [23,29–31]. While these findings are consistent with those presented here, each approach to ALK1 pathway inhibition is likely to have distinct effects in the clinical setting, and particularly when combined with different VEGF pathway inhibitors.

Preclinical studies of melanoma and breast cancer have shown that the anti-ALK1 monoclonal antibody (PF-03446962) as monotherapy did not show any significant anti-tumor efficacy but it did enhance tumor growth inhibition of a VEGFR TKI or bevacizumab [23]. The combination treatment was reported to diminish endothelial cell/pericyte co-staining in the xenograft tumors, however, the mechanism of the combination effect was not fully elucidated. Since ALK1 is expressed by activated endothelial cells, a direct antibody-mediated cytotoxicity towards ALK1 positive endothelial cells when combined with VEGF pathway inhibition could not be ruled out. Here we used a different strategy to block this pathway, BMP9/10 ligand trap, to show that simultaneous antagonism of ALK1 and VEGFR pathways resulted in downregulation of the Notch pathway. This leads to further decrease in tumor blood flow and durable growth inhibition in both sunitinib-naïve and sunitinib-refractory tumors. The combined dysregulation of VEGF/VEGFR, BMP9/ALK1 and Dll4/Notch signaling pathways can lead to a more profound effects on tumor angiogenesis delaying the development of acquired resistance to VEGFR TKI in RCC.

In a completed phase 1 study, dalantercept showed anti-tumor activity in patients with advanced cancer and a safety profile relatively distinct from inhibitors of the VEGF pathway [32]. Our data support the hypothesis that the combination of dalantercept and a VEGFR TKI can control growth of TKI-resistant RCC tumors. We anticipate similar results with the combination of axitinib with dalantercept due to the common mechanism of action between axitinib and sunitinib. Based on these findings, a multicenter randomized phase II study is currently underway exploring the combination of dalantercept plus axitinib vs axitinib plus placebo in patients with advanced RCC refractory to anti-VEGF therapy.

## MATERIALS AND METHODS

### Drug formulation and administration

Dalantercept (ALK1-Fc), and the control murine Fc protein were manufactured at Acceleron Pharma, Inc. and described elsewhere [11,12]. The recombinant proteins were diluted in TBS or PBS to 2 mg/ml and dosed intraperitoneally at 5 ml/kg three times a week. Sunitinib malate (clinical grade) was resuspended in citrate buffer (pH 2.3) and dosed at 40 mg/kg given by oral gavage daily, 6/7 days per week.

### Cell culture

The human VHL deficient human RCC cell lines, 786-O and A498 were obtained from the American Type Culture Collection (ATCC, Manassas, VA) and cultured for less than one month in aliquots and then frozen. Freshly thawed aliquots were used for each experiment. A498 cells were grown in Eagle's Minimum Essential Medium (EMEM). 786-O cells were cultured in RPMI 1640 medium (Cellgro).

### Tumor xenograft studies

For subcutaneous xenograft tumor models, female athymic nude/beige mice (Charles River Laboratories) or athymic nude mice (Harlan Laboratories) were used. The mice were housed and maintained in laminar flow cabinets under specific pathogen-free conditions. All experiments were approved by the Institutional Animal Care and Use Committee (IACUC) at Beth Israel Deaconess Medical Center or the local IACUC at Acceleron Pharma, Inc.

To establish RCC tumor xenografts, 786-O and A498 tumor cells were injected subcutaneously (10^7^ cells) into the flanks of 6–8 week old mice (~20 g on average). When tumors reached 12 mm along the long axis (~500 mm^3^ in volume), mice were randomized into treatment groups (5–8 mice per group). Treatment was continued until tumors reached 20 mm along the long axis or until 50 days after treatment initiation.

### Tumor blood flow imaging

Tumor blood flow imaging with arterial spin-labeled MRI (ASL MRI) was performed as previously described [5,33,34]. Briefly, mice were anesthetized and imaged with a 3-cm surface coil on a 3.0 T whole-body clinical MRI scanner. A single transverse slice of ASL imaging was carefully positioned at the center of the tumor, which was marked on the skin with a permanent marker pen for follow-up MRI studies. ASL images were obtained with a single-shot fast spin echo sequence by using a background suppressed and flow-sensitive alternating inversion-recovery strategy. Using standard methods to quantify tumor blood flow [35], a region of interest was drawn around the peripheral margin of the tumor on the reference image that was then copied to the blood flow image. The mean blood flow for the tumor tissue within the region of interest was derived.

### 3D tumor vascular network imaging and analysis

Micro-computed tomography (μCT) vascular imaging was used to compare tumor vascular networks between drug-treated and control groups. Animals were anesthetized with isoflurane and perfused with a solution of heparinized-saline (1000 unit/ml) followed by perfusion with Microfil (Flow Tech Inc., Carver, MA, USA), a polymerizing silicone rubber contrast agent. Perfused animals were sacrificed and kept at 4°C overnight to allow polymerization. The following day tumors were resected and fixed in 10% formalin and sent to SCANCO Medical for μCT imaging and subsequent image analysis. All samples were scanned on a high-resolution, volumetric μCT scanner (μCT40, SCANCO Medical, Zurich, Switzerland) using the following parameters: 10 μm isotropic voxel resolution, 300 ms exposure time, 2000 views, and 3 frames per view. The μCT-generated DICOM files were were combined into an image volume using the Teem (http://teem.sourceforge.net/) software package. VHLab (SCANCO Medical) was used for segmenting out the vessels from the imaging data and used for volume measurements. SCIRun (SCI Institute) was used to generate 3D renderings as well as for calculating the total vascular surface area. SCANCO Medical's software tools were used for evaluating vascular radius [36].

### Immunohistochemistry

Formalin-fixed paraffin-embedded (FFPE) tissue blocks from primary and metastatic clear cell RCC (ccRCC) were retrieved from Beth Israel Deaconess Medical Center under Dana Farber/ HCC IRB approved protocol 01-130. 12 primary ccRCCs were retrieved and one primary ccRCC was paired with a corresponding lung metastasis. Analysis was performed in selected areas of primary tumors containing low (G1-G2) and high (G3-G4) Fuhrman nuclear grade (FNG). Immunohistochemistry was performed on four micron-thick, FFPE tumor sections, which were initially deparaffinized, rehydrated and heated with a pressure cooker to 125°C for 30 seconds in citrate buffer for antigen retrieval and then incubated with peroxidase (Dako #S2003, Carpinteria, CA) and protein blocking reagents (Dako #X0909) respectively for 5 minutes. Sections were then incubated with anti-BMP-9 antibody (AbD Serotec #1406-1460) at 1:2000 dilution for 1 hour at room temperature followed by incubation with the Dako EnVision+ System HRP labeled polymer anti-rabbit (Dako #K4011) for 30 minutes. All sections were developed using the DAB chromogen kit (Dako K3468) for 2 minutes and then lightly counterstained with hematoxylin. The assay was validated using CHO cells that over express human BMP9 (generated at Acceleron Pharma, Inc.) and the parental CHO cell line. The presence of tumor cells with cytoplasmic staining was assessed. A case was considered positive if any positivity was detected. Immunohistochemistry of CD34 was performed on adjacent four-micron thick FFPE sections from sunitinib and ACE041, sunitinib only and vehicle treated xenografts as previously [37].

### Transcriptome profiling using RNA quantification sequencing

Mice harboring A498 tumors were treated with vehicle (n=3), sunitinib (n=4), dalantercept (n=3) or a combination of sunitinib and dalantercept (n=4) and tumors were harvested for RNA preparation at 30 days or when tumors reached 20mm in long axis. Transcriptome profiles of the tumors were generated using next-generation paired-end RNA sequencing.

Sequencing libraries were generated from double-stranded cDNA using the Illumina TruSeq kit according to the manufacturer's protocol for paired-end sequencing. Library quality was checked using the Agilent DNA High Sensitivity Chip and qRT-PCR. High quality libraries were sequenced on an Illumina HiSeq 2000. To achieve comprehensive coverage for each sample, we generated ~35-50 million paired end reads.

### RNA-Seq data analysis

The raw sequencing data was processed to remove adaptor, PCR primers and low quality transcripts using FASTQC and Trimomatic softwares. These high quality, clean reads were aligned against tumor (human, hg19) and host (mouse, mm10) genomes using tophat2 and bowtie2 software packages (http://tophat.cbcb.umd.edu/) [38]. Gene expression measurement was performed from aligned reads by counting the unique reads. The count data were preprocessed to remove all low expressing genes on the basis of counts per million (CPM) in samples (i.e. genes with CPM <1 in all samples). The read count based on gene expression data was normalized on the basis of library complexity and gene expression variation. The normalized data was compared among groups using a negative binomial model to identify differentially expressed genes [39]. The differentially expressed genes were identified on the basis of multiple test corrected P values (i.e. FDR,5%) (27) and fold changes (±2). Comparative analysis of gene lists with external primary tumor angiogenesis signature [40] was performed through Venn diagrams. Unsupervised analysis was performed on normalized and preprocessed count data using Principal Component Analysis (PCA) [41]. For comparison of differentially expressed mouse and human genes, we performed orthologous gene identification analysis. We identified human orthologs of all differentially expressed mouse genes using NCBI homology database [42].

### Pathway and regulatory molecules enrichment analysis

Ingenuity Pathway Analysis (IPA 8.0, Qiagen) was used to identify the pathways that are significantly affected by differentially expressed genes from different pairwise comparisons. The knowledge base of this software consists of functions, pathways and network models derived by systematically exploring the peer reviewed scientific literature. It calculates a p-value for each pathway according to the fit of users' data to the IPA database using one-tailed Fisher exact test. The pathways with multiple test corrected p-values <0.01 were considered significantly affected.

The regulatory module analysis was used to identify the cascade of upstream transcriptional regulators in the treatment groups [43]. The significance of this analysis was determined using one-tailed Fisher exact test [44].

### RT-PCR analysis

PCR primers were designed using the Primer 3 software from NCBI [45] and synthesized by Integrated DNA Technologies. PCR was performed as described previously [46]. PCR reactions for each sample were performed in duplicate, and copy numbers were measured as described previously [47]. The level of target gene expression was normalized against 18S rRNA. For some genes instead of absolute expression estimation we have performed relative quantitation approach to determine the expression relative to 18S expression [48].

### Statistical analysis

Statistical analysis of the data was done using GraphPad Prism 5 software (GraphPad Software Inc., La Jolla, CA) or Statistical functions developed using R language [49].

## SUPPLEMENTARY FIGURES


