# Supplementary material for: Inhibition of ALK1 signaling with dalantercept combined with VEGFR TKI leads to tumor stasis in renal cell carcinoma
Source: Oncotarget. 2016 May 26;7(27):41857–69. doi: 10.18632/oncotarget.9621 (PMC5173101; doi:10.18632/oncotarget.9621)
Supplement: Supplementary file 1 [file oncotarget-07-41857-s001.pdf]

Inhibition of ALK1 signaling with dalantercept combined with VEGFR TKI leads to tumor stasis in renal cell carcinoma

SUPPLEMENTARY FIGURES

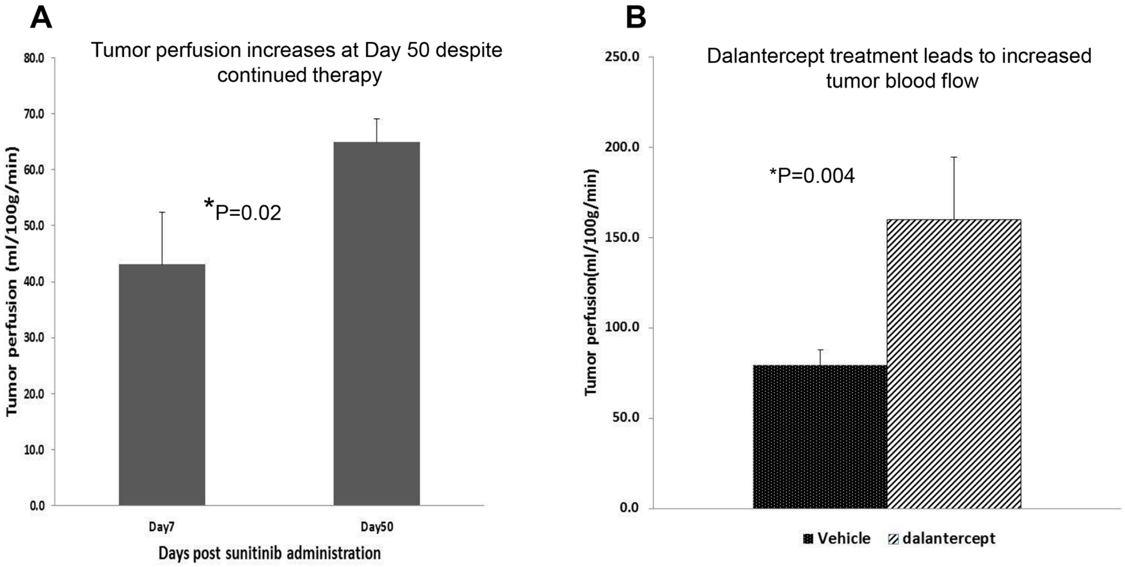

Supplementary Figure S1: Tumor blood flow resumes with sunitinib therapy and increases with dalantercept monotherapy after prolonged treatment. A. Tumor blood flow increases at day 50 compared to day 7 in A498 tumor model despite continued sunitinib therapy. **B.** Dalantercept treatment leads to increased tumor blood flow compared to vehicle treatment at day 48.

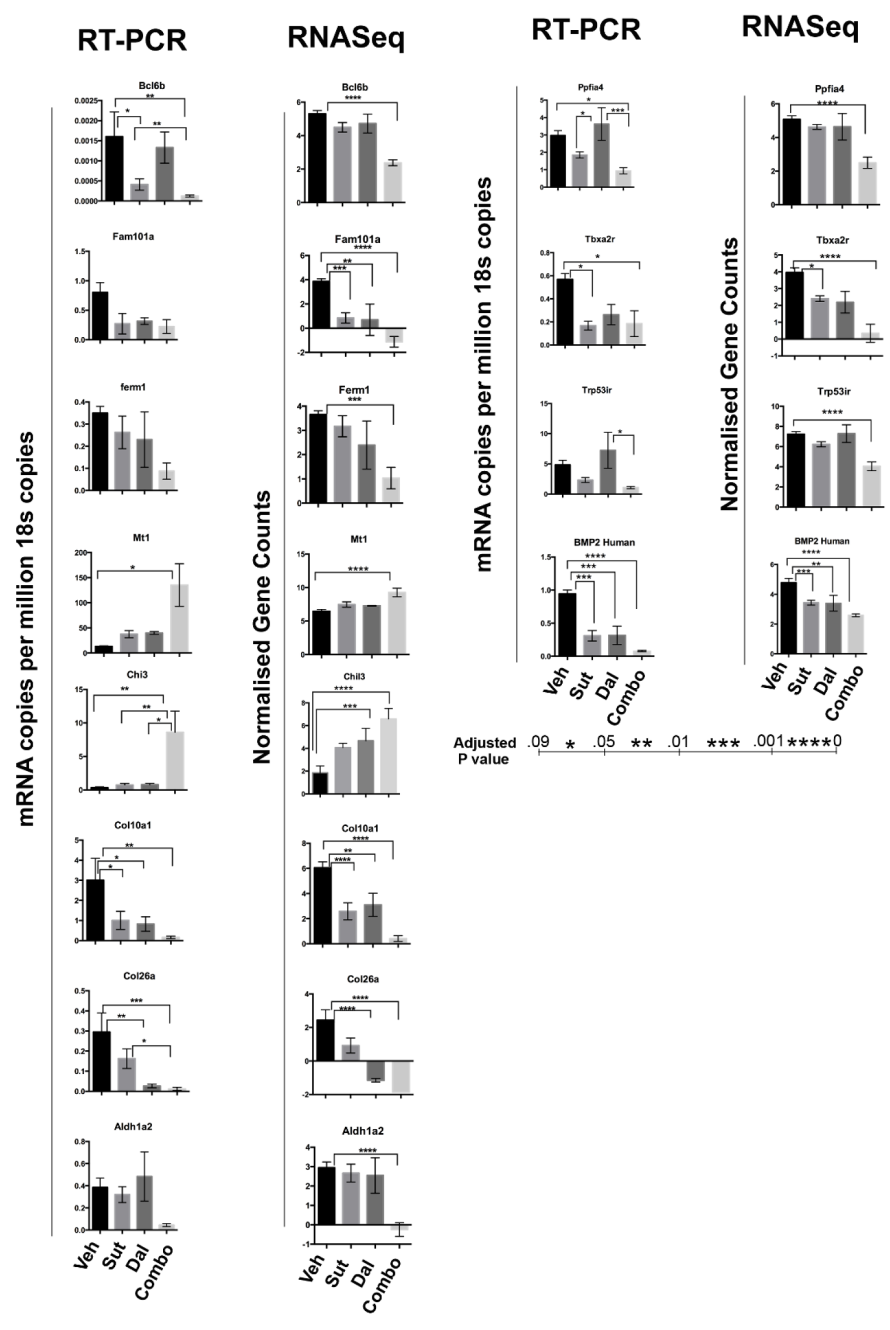

Supplementary Figure S2: Validation of differentially expressed Host (Murine) and Tumor (Human) genes using RT-PCR. We validated the expression changes in the RNA-Seq analysis of 1 tumor and 11 host genes using RT-PCR. Significant correlation was observed between RT-PCR and RNA-Seq based expression profile for the majority of the genes. RT-PCR was performed on vehicle, sunitinib, dalantercept and combination treated tumors with at least two tumors per arm (n=2-3 tumors per arm). Graph shows mean ± SEM of relative mRNA levels after normalization to 18S copies. For RNA-Seq statistical significance was determined using multiple test corrected P value after negative binomial analysis. For RT-PCR data, statistical significance was determined by calculating Tukey's t test adjusted P value.

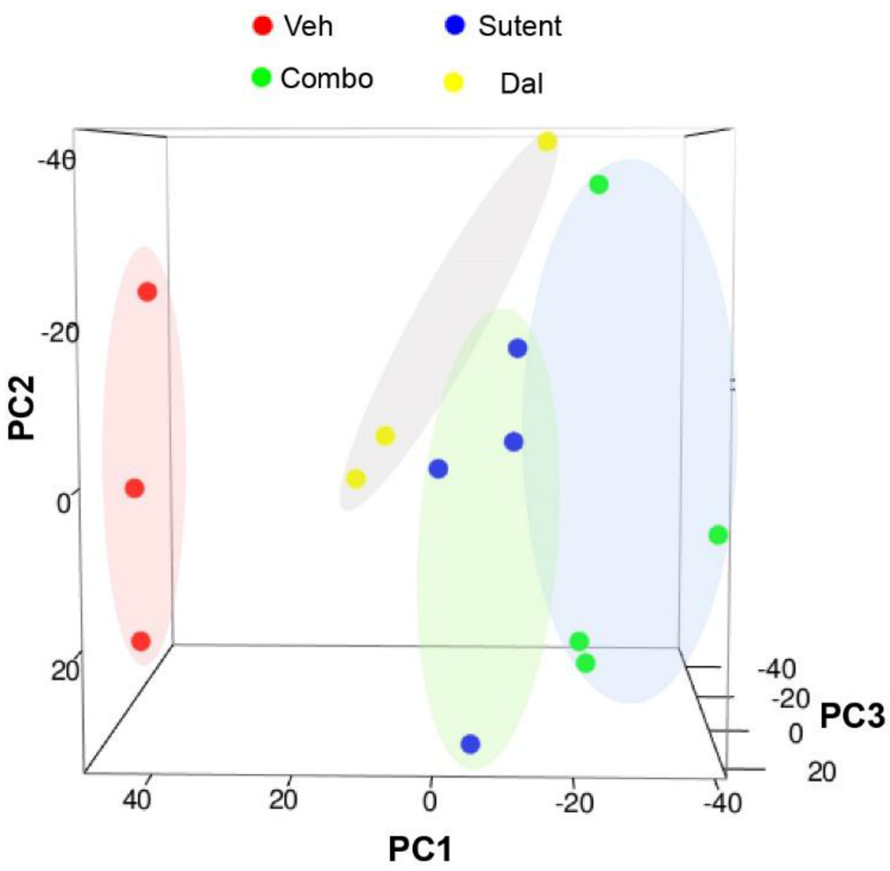

Supplementary Figure S3: Principal Component Analysis (PCA) of normalized and pre-processed RNA-Seq data of vehicle, sunitinib, dalantercept and combination therapy treated xenograft tumors. The first component with highest variance is shown on the X-axis separating untreated samples from treated samples (i.e. sunitinib, dalantercept and combination therapy). The analysis depicted maximum transcriptional differences between combination therapy and vehicle treated samples. The analysis also depicted transcriptional variation between combination therapy samples compared to single therapy samples.
